# Trichostatin A effects on gene expression in the protozoan parasite *Entamoeba histolytica*

**DOI:** 10.1186/1471-2164-8-216

**Published:** 2007-07-05

**Authors:** Gretchen M Ehrenkaufer, Daniel J Eichinger, Upinder Singh

**Affiliations:** 1 Department of Microbiology and Immunology, Stanford University School of Medicine, Stanford, California, 94305-5107, USA; 2 Department of Medical Parasitology, New York University School of Medicine, 550 First Avenue, New York, NY 10016, USA; 3 Division of Infectious Diseases, Department of Internal Medicine, S-143 Grant Building, 300 Pasteur Drive, Stanford, CA, 94305, USA

## Abstract

**Background:**

Histone modification regulates chromatin structure and influences gene expression associated with diverse biological functions including cellular differentiation, cancer, maintenance of genome architecture, and pathogen virulence. In *Entamoeba*, a deep-branching eukaryote, short chain fatty acids (SCFA) affect histone acetylation and parasite development. Additionally, a number of active histone modifying enzymes have been identified in the parasite genome. However, the overall extent of gene regulation tied to histone acetylation is not known.

**Results:**

In order to identify the genome-wide effects of histone acetylation in regulating *E. histolytica *gene expression, we used whole-genome expression profiling of parasites treated with SCFA and Trichostatin A (TSA). Despite significant changes in histone acetylation patterns, exposure of parasites to SCFA resulted in minimal transcriptional changes (11 out of 9,435 genes transcriptionally regulated). In contrast, exposure to TSA, a more specific inhibitor of histone deacetylases, significantly affected transcription of 163 genes (122 genes upregulated and 41 genes downregulated). Genes modulated by TSA were not regulated by treatment with 5-Azacytidine, an inhibitor of DNA-methyltransferase, indicating that in *E. histolytica *the crosstalk between DNA methylation and histone modification is not substantial. However, the set of genes regulated by TSA overlapped substantially with genes regulated during parasite development: 73/122 genes upregulated by TSA exposure were upregulated in *E. histolytica *cysts (p-value = 6 × 10^-53^) and 15/41 genes downregulated by TSA exposure were downregulated in *E. histolytica *cysts (p-value = 3 × 10^-7^).

**Conclusion:**

This work represents the first genome-wide analysis of histone acetylation and its effects on gene expression in *E. histolytica*. The data indicate that SCFAs, despite their ability to influence histone acetylation, have minimal effects on gene transcription in cultured parasites. In contrast, the effect of TSA on *E. histolytica *gene expression is more substantial and includes genes involved in the encystation pathway. These observations will allow further dissection of the effects of histone acetylation and the genetic pathways regulating stage conversion in this pathogenic parasite.

## Background

Regulation of gene expression is a complex process controlled by sequence-specific DNA binding proteins, modulation of chromatin structure, and post-transcriptional modifications. In recent years, increased attention has been given to the role of epigenetic mechanisms, such as the modification of histone proteins, in gene regulation [1]. These modifications, including methylation, phosphorylation and acetylation, occur at specific amino acids on the N-terminal tails of histone core proteins, particularly H3 and H4, and regulate chromatin structure and gene expression [2,3]. Methylation of histones at lysine residues has typically been associated with transcriptionally silent heterochromatin [4]. In contrast, lysine acetylation is generally thought to trigger the opening of chromatin structure and transcriptional activation [5,6]. However, this is an oversimplified model and does not represent the true complexity of these processes, which can also differ between lower and higher eukaryotes [7]. Individual modifications of histones may be interdependent, with methylation of certain lysine residues blocking or enhancing the addition of acetyl groups nearby [8,9]. In addition, methylation of arginine residues may actually activate the transcription of some genes. A number of proteins have been identified which regulate these modifications, including histone acetyltransferases (HATs), histone deacetylases (HDACs), histone methyltransferases (HMT), and a recently discovered class of histone demethylases [10].

The protozoan parasite *Entamoeba histolytica *has two morphologically distinct life cycle forms, the infectious cyst form that transmits disease from person to person, and the trophozoite form that multiplies in the colon and eventually differentiates back into the cyst form. While in the colon, the trophozoite form causes invasive disease (colitis and liver abscess) in 50 million people per year making amebiasis a leading parasitic cause of death worldwide [11]. Despite its importance for human health, little is known about how this parasite modulates its gene expression during host invasion or conversion from one life cycle form to the other. Changes in transcript abundance in *E. histolytica *are associated with host invasion [12], with exposure to oxidative stress [13], and with conversion between the cyst and trophozoite forms [14], but the mechanisms regulating transcript levels are poorly understood. A number of amebic promoter elements and transcription factors have been described [15] and DNA methylation has been identified as playing a role in controlling a limited amount of amebic gene expression [16,17]. Functional histone-modifying enzymes, such as HATs of the MYST and GNAT families, and a Class I HDAC, and acetylated histones have been described in *E. histolytica *[18], but their activities have not yet been tied to gene expression changes.

In *Entamoeba invadens*, a parasite of reptiles, a role for histone modifications in the regulation of stage conversion has been proposed. Histones of *in vitro *cultured *E. invadens *trophozoites are constitutively acetylated, with the levels of acetylation increasing in the presence of Trichostatin A (TSA), but decreasing in the presence short chain fatty acids (SCFA) such as butyrate [19]. The decreased histone acetylation resulting from butyrate exposure was unexpected, as this compound induces increased histone acetylation in all other eukaryotic cells in which it has been examined [20-22]. Treatment of *E. invadens *trophozoites with TSA or SCFAs blocks their *in vitro *development to the cyst stage, suggesting a biological role for histone modification in *Entamoeba *development [23]. The link between cyst development and histone acetylation observed in *E. invadens *has not been recapitulated in *E. histolytica *due to lack of an *in vitro *system for encystation. Complicating the studies of *E. histolytica *is the fact that individual laboratory strains of the parasite have different baseline histone acetylation patterns [19]. For example, *E. histolytica *HM-1:IMSS under standard culture conditions does not have any detectable acetylated H4, whereas two other strains, *E. histolytica *Rahman and *E. histolytica *200:NIH, have multiply-acetylated H4 populations under the same growth conditions. Additionally, both of these strains shift to a hyperacetylated H4 pattern when treated with TSA. Furthermore, when grown with SCFAs, *E. histolytica *Rahman and *E. histolytica *200:NIH H4 histones become hypoacetylated, similar to the response of *E. invadens*. The unusual hypoacetylation response to butyrate of *Entamoeba *suggests that SCFAs regulate histone acetylation and gene expression in a unique way, one that most likely reflects parasite adaptation to growth in the presence of the large amounts of the short chain fatty acids found in the colon.

In other protozoan parasites histone modification plays important roles in life cycle progression and antigenic variation. In *Toxoplasma gondii*, chromatin immunoprecipitation analysis has demonstrated differential acetylation and methylation in the promoters of stage-specific genes during stage conversion [24]. In addition, treatment with drugs that affect histone acetylation or arginine methylation affected both stage-conversion and overall gene expression [24,25]. In *Plasmodium falciparum *histone H4 acetylation states and promoter occupation by the Sir2 transcriptional regulator have been linked to changes in the expression of *var *genes [26].

To gain insights into the role of histone acetylation in regulating gene expression in *E. histolytica *we treated *E. histolytica *trophozoites with SCFA or TSA and performed whole genome transcriptional profiling. The data revealed that in *E. histolytica *there was minimal transcriptional response to SCFAs, with ~0.1% of genes modulated ± 2-fold. In contrast, the transcriptional response to TSA was greater (~2% of genes modulated ± 2-fold), and the gene expression changes overlapped significantly with the transcriptional signature of the developmental pathway to cysts [14]. This work represents the first genome wide analysis of transcriptional changes associated with histone modifications in *E. histolytica *and reveals a subset of developmentally regulated genes whose expression correlates with changes in the level of histone acetylation.

## Results

### *E. histolytica *strains HM-1:IMSS, Rahman, and 200:NIH have similar but not identical expression profiles of genes encoding histone-modifying enzymes

To account for differences in levels of histone acetylation between *E. histolytica *strains, we analyzed previously published data from a whole genome microarray to compare the gene expression profiles of three strains of *E. histolytica *(HM-1:IMSS, Rahman and 200:NIH) [14]. Overall, the expression profiles of the three *E. histolytica *strains were highly similar although some genes whose transcript levels were significantly different between strains (± 2-fold and p-value <0.05) were identified. Overall, 127 genes had higher expression in *E. histolytica *HM-1:IMSS, 261 genes had higher expression in *E. histolytica *200:NIH, and 71 genes had higher expression in *E. histolytica *Rahman compared to the other two *E. histolytica *strains (Additional File 1). For our purposes, we focused on the expression levels of genes involved in the regulation of histone acetylation and chromatin structure. Surprisingly, given the absence of multiply acetylated histones in *E. histolytica *HM-1:IMSS, several HAT genes (2.m00560 and 67.m00100) were expressed at relatively high levels in HM-1:IMSS trophozoites (Additional File 2). Some differences in expression levels of histone modification genes between the strains were identified. One HAT (100.m00145) had significantly higher expression *E. histolytica *200:NIH. Two Sir2 family HDAC genes were expressed differentially between strains: 251.m00088 was expressed at significantly higher levels in *E. histolytica *HM-1:IMSS and 2.m00521 was more highly expressed in *E. histolytica *200:NIH. The overexpression of particular Sir2 genes in yeast leads to global histone deacetylation [6]. The increased expression of a HAT gene in *E. histolytica *200:NIH trophozoites and high expression of a Sir2 HDAC gene in *E. histolytica *HM1:IMSS is consistent with the histone acetylation patterns in these strains. However, the actual levels histone proteins and their relative enzyme activities in these parasite strains will need to be established before conclusions can be made about the causes of the differences in levels of multiply-acetylated histones in the isolates.

### Growth of *E. histolytica *in the presence of SCFA has minimal effects on parasite gene expression

SCFA have substantial effects on histone acetylation and development in *Entamoeba *[19,23]. To determine the effect of SCFA on amebic growth, we tested the ability of *E. histolytica *to grow in TYI-S-33 LG medium ± SCFA. In media with SCFA, there were no significant alterations in growth of *E. histolytica *HM-1:IMSS and 200:NIH parasites at 16 hours, the time points at which the microarray experiments were performed (data not shown). To determine if growth with SCFA altered parasite gene expression, we compared the transcriptional profile of *E. histolytica *200:NIH trophozoites grown in TYI-S-33 LG medium ± SCFA. The *E. histolytica *200:NIH strain was used for this and subsequent microarray analyses because it has an histone acetylation pattern similar to encystation-competent *E. invadens *and hence was the most likely to provide insights into developmental pathways regulated by histone acetylation [19]. For each strain and culture condition the number of arrays and the correlations between array data sets are outlined in Tables 1 and 2. Genes were considered differentially expressed if they had a ≥ 2-fold change and were significant with an FDR of < 0.05. Overall, few changes in gene expression were observed when we compared *E. histolytica *200:NIH grown in TYI-S-33 or LG medium to those grown in LG medium with SCFA. Only 11 genes were differentially regulated by addition of SCFA, and there were no changes in genes associated with histone modifications (Table 3; Additional File 3). Thus, although growth of *E. histolytica *200:NIH trophozoites with SCFAs results in hypoacetylation of H4 histones [19], these changes are apparently not associated with significant alterations in the gene expression profile of axenically grown trophozoites.

**Table 1:** An overview of the microarrays generated and used in the analysis.

	***E. histolytica*** **200:NIH**	***E. histolytica*** **200:NIH**	***E. histolytica*** **200:NIH**
**Number of arrays**	4	3	3
**Minimum correlation of the arrays in condition**	0.96	0.96	0.98
**Culture medium**	TYI-S-33 or LG	LG+SCFA	LG+TSA

Summary of the culture conditions and number of arrays performed for each condition with *E. histolytica *200:NIH.

**Table 2:** Correlations of arrays used in analysis.

	***E. histolytica*** **200:NIH** **(TYI-S-33 or LG)**	***E. histolytica*** **200:NIH** **(LG+SCFA)**	***E. histolytica*** **200:NIH** **(LG+TSA)**
**200:NIH (TYI-S-33 or LG)**	X	0.99	0.98
**200:NIH (LG+SCFA)**	X	X	0.98
**200:NIH (LG+TSA)**	X	X	X

The average normalized microarray data of a given condition was compared to another condition and the correlations are shown.

**Table 3:** *E. histolytica *genes regulated by exposure short-chain fatty acids.

**Probe ID**	**GenBank ID**	**Description**	**Fold-change**	**FDR**
**Up-regulated**				
122.m00139_at	XM_647238	ADP-ribosylation factor, putative	2.11	1.97E-02
14.m00310_at	XM_651236	hypothetical protein	6.70	2.09E-03
205.m00100_s_at	XM_645582	hypothetical protein	2.57	2.86E-02
22.m00285_at	XM_650728	hypothetical protein	19.36	1.19E-11
295.m00030_at	XM_644416	conserved hypothetical protein	20.75	3.12E-14
418.m00028_at	XM_643537	70 kDa heat shock protein, putative	6.03	1.97E-02
522.m00017_at	XM_643097	hypothetical protein	3.41	6.42E-03
522.m00018_x_at	XM_643100	hypothetical protein	2.78	1.19E-03
585.m00015_s_at	XM_642978	conserved hypothetical protein	3.16	1.82E-03
72.m00179_at	XM_648717	hypothetical protein	6.25	5.69E-04
**Down-regulated**				
5.m00482_at	XM_651923	protein kinase, putative	-2.82	2.86E-02

The mean trophozoite expression value for *E. histolytica *200:NIH strain under standard culture conditions, the fold-change in SCFA treated parasites, FDR, GenBank ID number, and gene annotations are shown. Ten genes are upregulated and one is downregulated. 205.m00100_s_at also represents 105.m00129.

### Growth of *E. histolytica *200:NIH in the presence of TSA changes the amebic transcriptional profile

**Figure 1:**
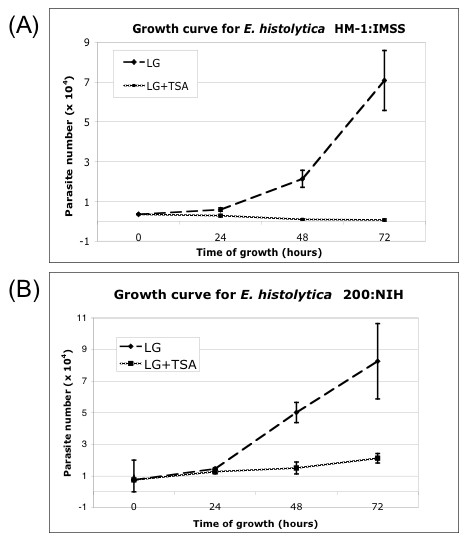
**Growth rates of *E. histolytica *HM-1:IMSS and 200:NIH in medium with Trichostatin A**. Log-phase trophozoites (7,500 cells) were seeded into 14 ml tubes containing fresh media (LG or LG+TSA). Aliquots were counted every 24 hours. **(A) ***E. histolytica *HM1:IMSS parasites stopped proliferating immediately upon transfer to LG+TSA containing media. **(B) ***E. histolytica *200:NIH can grow in LG+TSA, although at a reduced rate compared to growth in LG medium. Experiments were performed a minimum of two times and standard deviation is shown.

To determine the effect of TSA on amebic growth, *E. histolytica *strains HM-1:IMSS and 200:NIH were grown in TYI-S-33 LG medium ± TSA. *E. histolytica *HM-1:IMSS trophozoites died within 2–3 days in medium with 150 nM TSA, whereas *E. histolytica *200:NIH survived and grew but at a much slower rate than in TYI-S-33 LG medium alone (Figure 1). As treatment with TSA has been shown to cause histone H4 hyperacetylation [19], the effects of TSA on parasite gene expression were determined by comparing the transcriptional profile of *E. histolytica *200:NIH trophozoites grown in TYI-S-33 LG medium ± TSA. Four microarrays were hybridized with RNA from *E. histolytica *200:NIH parasites grown in TYI-S-33 or LG medium and compared to three microarrays hybridized with RNA from *E. histolytica *200:NIH parasites grown in LG medium plus TSA for 16–72 hours. In contrast to the minimal transcriptional changes seen in the SCFA treatment, TSA exposure resulted in significant changes in gene expression. Overall, 163 genes, ~2% of the genes tested, showed altered transcript abundance, with 122 genes upregulated and 41 genes downregulated by TSA exposure (Tables 4 and 5; Additional File 4). Of the 122 genes whose expression increased with TSA treatment, 46 (38%) had normalized expression < 0.2 in 200:NIH trophozoites grown in TYI-S-33 or LG, indicating that they may be silenced under normal *in vitro *growth conditions.

**Table 4:** Subset of *E. histolytica *genes upregulated by exposure to Trichostatin A.

**Probe ID**	**GenBank ID**	**Description**	**Fold-change**	**FDR**
489.m00024_at	XM_643195	*hypothetical protein*	39.96	7.60E-16
**14.m00310_at**	XM_651236	*hypothetical protein*	39.67	2.32E-20
295.m00030_at	XM_644416	conserved hypothetical protein	38.77	2.29E-20
522.m00017_at	XM_643097	hypothetical protein	20.45	4.53E-15
418.m00028_at	XM_643537	*70 kDa heat shock protein, putative*	17.45	6.71E-10
36.m00204_s_at	XM_650008	hypothetical protein	14.84	1.22E-08
451.m00037_s_at	XM_643383	hypothetical protein	14.68	3.96E-04
556.m00022_x_at	XM_643031	hypothetical protein	14.37	5.75E-07
12.m00306_at	XM_651386	*hypothetical protein*	13.25	1.64E-05
376.m00054_s_at	XM_643784	hypothetical protein	13.02	1.62E-10
564.m00020_x_at	XM_643013	hypothetical protein	12.93	8.19E-10
82.m00164_s_at	XM_648353	hypothetical protein	12.88	8.00E-12
28.m00298_s_at	XM_650406	hypothetical protein	11.79	5.97E-09
110.m00118_at	XM_647568	Rho family GTPase	11.60	2.26E-14
50.m00195_s_at	XM_649449	hypothetical protein	11.36	3.02E-05
135.m00094_at	XM_646923	hypothetical protein	10.98	2.38E-08
493.m00030_x_at	XM_643175	hypothetical protein	10.25	1.40E-24
**337.m00049_at**	XM_644075	*hypothetical protein*	9.93	5.43E-07
164.m00105_x_at	XM_646368	hypothetical protein	9.92	4.32E-05
847.m00011_x_at	XM_642805	hypothetical protein	9.63	9.76E-05
205.m00100_s_at	XM_645582	hypothetical protein	9.48	3.01E-08
373.m00052_at	XM_643804	hypothetical protein	8.71	1.14E-06
395.m00030_x_at	XM_643673	*protein kinase, putative*	8.68	6.64E-04
749.m00013_s_at	XM_642848	hypothetical protein	8.61	1.85E-03
227.m00077_at	XM_645230	*hypothetical protein*	8.40	1.70E-08
728.m00012_x_at	XM_642855	hypothetical protein	8.37	5.83E-06
460.m00025_x_at	XM_643328	hypothetical protein	8.00	3.05E-02
167.m00116_x_at	XM_646306	hypothetical protein	7.98	3.77E-03
621.m00019_at	XM_642928	hypothetical protein	7.84	8.57E-06
584.m00019_at	XM_642979	*heat shock protein 70, putative*	7.82	5.54E-06
477.m00021_at	XM_643239	hypothetical protein	7.60	1.27E-03
123.m00123_x_at	XM_647209	conserved hypothetical protein	7.48	3.84E-05
76.m00146_at	XM_648554	hypothetical protein	7.34	1.75E-04
89.m00125_s_at	XM_648139	hypothetical protein	7.32	2.51E-05
220.m00068_x_at	XM_645332	hypothetical protein	7.14	2.48E-03
263.m00053_at	XM_644795	hypothetical protein	6.95	2.56E-04
804.m00006_x_at	XM_642821	hypothetical protein	6.94	2.44E-04
72.m00179_at	XM_648717	hypothetical protein	6.85	1.17E-04
211.m00072_at	XM_645468	zinc finger protein, putative	6.80	5.04E-03
102.m00082_at	XM_647784	*hypothetical protein*	6.74	6.19E-03
411.m00025_x_at	XM_643570	*hypothetical protein*	6.68	8.26E-03
162.m00085_at	XM_646412	predicted protein	6.61	3.81E-06
157.m00087_x_at	XM_646502	conserved hypothetical protein	6.50	8.44E-04
12.m00326_at	XM_651342	*hypothetical protein*	6.42	4.10E-04
52.m00169_x_at	XM_649399	hypothetical protein	6.39	2.21E-03
22.m00288_at	XM_650731	*hypothetical protein*	6.28	6.49E-04
71.m00129_at	XM_648728	hypothetical protein	6.16	5.70E-05
6.m00428_at	XM_651814	hypothetical protein	6.10	1.46E-04
496.m00027_x_at	XM_643167	*hypothetical protein*	5.98	9.24E-06
**135.m00113_at**	XM_646942	*hypothetical protein*	5.91	4.26E-03

The mean trophozoite expression value for *E. histolytica *200:NIH strain under standard culture conditions, the fold-change in TSA treated parasites, FDR, GenBank ID number, and gene annotations are shown. Genes in bold have been confirmed by RT-PCR. Genes regulated during trophozoite to cyst development are shown in italics. Additional loci represented by crosshybridizing _s_at probe sets are as follows: 36.m00204_s_at: 88.m00180; 451.m00037_s_at: 467.m00031 and 50.m00196; 376.m00054_s_at: 116.m00123; 82.m00164_s_at: 82.m00157; 28.m00298_s_at: 481.m00033 and 90.m00158; 205.m00100_s_at: 105.m00129; 749.m00013_s_at: 142.m00151; 89.m00125_s_at: 357.m00038.

**Table 5:** *E. histolytica *genes downregulated by exposure to Trichostatin A.

**Probe ID**	**GenBank ID**	**Description**	**Fold-change**	**FDR**
111.m00118_at	XM_647540	leishmaniolysin-related peptidase, putative	-44.44	0.00E+00
233.m00105_at	XM_645153	hypothetical protein	-29.24	0.00E+00
7.m00429_at	XM_651789	Beige BEACH domain protein, putative	-21.88	8.51E-10
**223.m00071_at**	XM_645286	*hypothetical protein*	-10.88	3.04E-04
31.m00224_at	XM_650243	hypothetical protein	-9.43	2.76E-03
**1.m00712_at**	XM_652408	hypothetical protein	-8.62	1.64E-05
**223.m00075_at**	XM_645290	*lipid phosphatase, putative*	-6.71	1.34E-03
105.m00133_at	XM_647680	NADP-dependent alcohol dehydrogenase	-5.81	8.19E-05
223.m00077_x_at	XM_645292	hypothetical protein	-5.65	8.47E-03
585.m00015_s_at	XM_642978	conserved hypothetical protein	-5.52	1.11E-02
223.m00069_at	XM_645284	*hypothetical protein*	-5.49	9.05E-03
223.m00068_at	XM_645283	*scavenger mRNA decapping enzyme, putative*	-5.24	2.27E-03
17.m00307_s_at	XM_651008	fatty acid elongase, putative	-5.08	1.34E-02
234.m00042_at	XM_645139	hypothetical protein	-5.00	2.34E-02
66.m00150_at	XM_648887	high mobility group protein, putative	-4.78	3.68E-02
131.m00139_at	XM_647034	M-phase inducer phosphatase, putative	-4.72	2.36E-02
249.m00083_at	XM_644976	hypothetical protein	-4.44	3.58E-03
7.m00436_at	XM_651745	*actobindin homolog, putative*	-4.31	7.67E-03
223.m00079_at	XM_645294	*cysteinyl-tRNA synthetase, putative*	-4.20	1.54E-02
425.m00057_s_at	XM_643497	conserved hypothetical protein	-4.10	2.14E-02
223.m00070_at	XM_645285	protein kinase, putative	-4.08	2.28E-02
4.m00636_at	NA	*pseudogene, galactose-specific adhesin light subunit*	-4.00	4.34E-02
217.m00081_s_at	XM_645388	hypothetical protein	-3.91	1.64E-02
92.m00148_s_at	XM_648072	conserved hypothetical protein	-3.88	4.66E-02
223.m00078_at	XM_645293	ribonuclease, putative	-3.85	3.89E-02
180.m00114_at	XM_646068	hypothetical protein	-3.73	1.75E-03
223.m00067_at	XM_645282	*integral membrane protein, putative*	-3.73	3.58E-03
223.m00074_at	XM_645289	*hypothetical protein*	-3.72	1.80E-02
223.m00076_at	XM_645291	hypothetical protein	-3.42	9.24E-03
10.m00362_at	XM_651510	cysteine proteinase, putative	-3.41	3.20E-09
54.m00183_at	XM_649345	hypothetical protein	-3.33	4.69E-02
242.m00078_s_at	XM_645064	*cysteine protease 1*	-2.89	3.47E-03
**77.m00173_at**	XM_648536	hypothetical protein	-2.76	2.82E-04
17.m00351_at	XM_651053	galactose-inhibitable lectin 35 kda subunit precursor	-2.75	2.84E-02
9.m00419_at	XM_651593	Fe-hydrogenase, putative	-2.72	1.73E-03
52.m00148_at	XM_649403	*lysozyme, putative*	-2.61	4.36E-02
154.m00120_at	XM_646541	glucosidase II alpha subunit, putative	-2.57	3.53E-02
9.m00416_at	XM_651590	*hypothetical protein*	-2.49	4.28E-02
47.m00182_at	XM_649559	fatty acid elongase, putative	-2.26	3.57E-02
22.m00269_s_at	XM_650745	hypothetical protein	-2.19	2.76E-02
77.m00178_s_at	XM_648541	*surface antigen ariel1-related*	-2.08	3.15E-02

The mean trophozoite expression value for *E. histolytica *200:NIH strain under standard culture conditions, the fold-change in TSA treated parasites, FDR, GenBank ID number, and gene annotations are shown. Genes in bold have been confirmed by RT-PCR. Genes regulated during trophozoite to cyst development are shown shaded in italics. Additional loci represented by crosshybridizing _s_at probe sets are as follows: 17.m00307_s_at: 17.m00304; 425.m00057_s_at: 10.m00394; 217.m00081_s_at: 20.m00277; 92.m00148_s_at: 250.m00108 and 284.m00087; 242.m00078_s_at: 79.m00156; 77.m00178_s_at: 19.m00343 and 533.m00017.

### Semi-quantitative RT-PCR confirmation of array results

**Figure 2:**
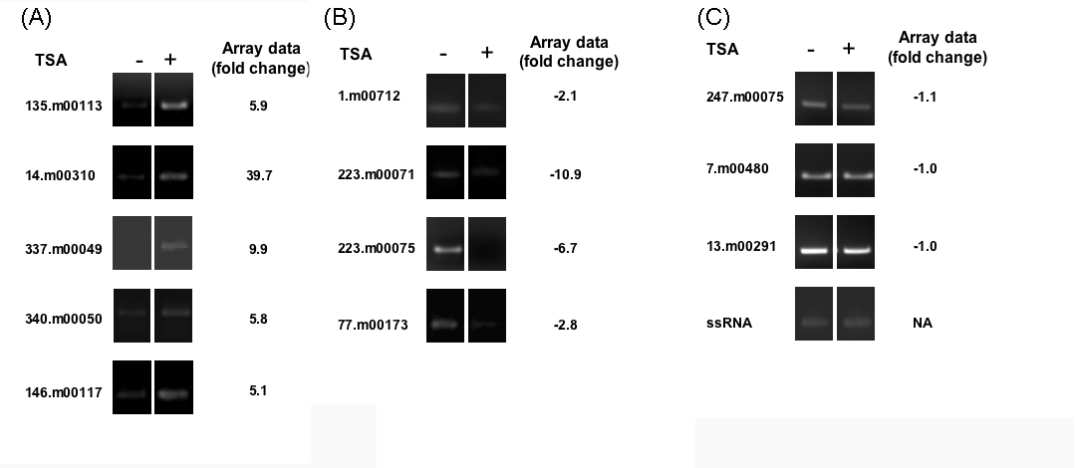
**RT-PCR confirms microarray data for genes regulated by TSA**. Genes found to be differentially expressed based on the array data were tested by semi-quantitative RT-PCR. RNA from log phase *E. histolytica *200:NIH trophozoites exposed to 150 nM TSA for 16 hours was used to generate cDNA for the analysis. **(A) **Genes identified by the array analysis as upregulated in TSA treated parasites (135.m00113, 14.m00410, 337.m00049, 340.m00050 and 146.m00117) are shown. **(B) **Genes identified as downregulated in TSA parasites (1.m00712, 223.m00071, 223.m00075, and 77.m00173) are shown. **(C) **Genes identified as being unchanged in TSA treated parasites (247.m00075 and 7.m00480, 13.m00291) and small subunit ribosomal RNA (X61116) were used as a loading control. For all genes, the trends indicated by the array data were recapitulated by the RT-PCR analysis. For all samples, a control reaction without reverse transcriptase control was performed, and was negative.

To confirm the expression patterns observed by microarray analysis, we performed semi-quantitative RT-PCR on 5 genes upregulated by exposure to TSA (135.m00113, 14.m00310, 337.m00049, 340.m00050 and 146.m00117), and 4 genes downregulated by exposure to TSA (1.m00712, 223.m00071, 223.m00075, and 77.m00173) (Figure 2). The gene for ssRNA, 247.m00075, 13.m00291 and 7.m00480 were used as loading controls. Serial dilutions of cDNA were performed for each sample. In all cases, RT-PCR results confirmed the array data.

### A substantial number genes regulated by TSA are also developmentally regulated in *E. histolytica*

**Figure 3:**
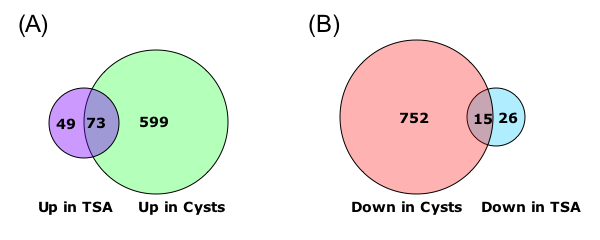
**Venn diagram of genes regulated by TSA and during stage conversion**. Overlap of genes regulated by TSA with developmentally regulated genes is shown. Of 122 genes upregulated by TSA treatment, 73 also show increased expression in cysts. Of the 41 genes downregulated by TSA treatment, 15 have increased expression in trophozoites. Both of these overlaps are statistically significant (p = 6 × 10^-53 ^and p = 3 × 10^-7 ^respectively).

We compared the lists of genes regulated by TSA to a number of transcriptional profiles previously generated with *E. histolytica *parasites. There was no significant overlap of genes modulated by TSA with parasite genes modulated in a mouse model of colitis [27] or after exposure to 5-Azacytidine [16]. However, the profile of genes regulated by TSA was found to overlap substantially with the profiles of genes differentially expressed in the two developmental stages (trophozoites and cysts) of *E. histolytica *[14] (Figure 3 and Tables 4 and 5). There was significant overlap between genes upregulated by TSA treatment and cyst-specific genes, with 73 of the 122 genes upregulated by TSA also upregulated in cysts (p-value = 6 × 10^-53^). Genes downregulated by TSA treatment overlapped significantly with trophozoite-specific genes, with 15 of the 41 genes downregulated by TSA also downregulated in cysts (p-value = 3 × 10^-7^). There was no significant overlap in the opposite direction (4 genes downregulated by TSA were upregulated in cysts and 4 genes upregulated by TSA were downregulated in cysts). Genes that were upregulated in both TSA-treated trophozoites and in cysts include some of the most highly induced genes under both conditions. An example is the hypothetical protein (489.m00024), which shows a ~40-fold increase in expression in TSA treated parasites and >500-fold increase in cysts [14]. Also included in this group are several genes encoding heat shock proteins (418.m00028 and 136.m00105) and putative signaling molecules (acid sphingomyelinase, 18.m00321 and a protein kinase, 395.m00030).

### Genes regulated in *E. histolytica *200:NIH by exposure to Trichostatin A

#### Heat shock proteins

A number of heat shock proteins, including Hsp70 isoforms (64.m00148, 584.m00019, 65.m00150 and 418.m00028) were induced by TSA treatment. Whether these genes are regulated by histone acetylation, or whether their induction is due to a stress response of the parasites to growth in TSA is unclear at this point. A gene expression response to heat shock was previously reported to be linked to encystation in *E. invadens *[28], thus high expression of these genes indeed appears to be characteristic of the transcriptional profile of stage conversion.

#### Signaling molecules

Genes regulated by treatment with TSA include several that are likely to have functions in signal transduction. These include protein kinases (14.m00339 and 395.m00030) and a Rho family GTPase (110.m00118), all with increased expression in TSA-treated parasites. A protein kinase (223.m00070) and a protein phosphatase (131.m00139) are both downregulated during TSA treatment. The regulation of these putative signaling molecules by TSA may suggest a role for histone acetylation in modulating signal transduction and responses to environmental factors in *E. histolytica*. Also upregulated by TSA are several genes, which could play a role in transcriptional regulation such as a Myb family protein (175.m00117 and zinc finger domain containing proteins (211.m00072 and 68.m00203).

#### Virulence

Several genes with roles in *E. histolytica *virulence were downregulated by TSA treatment. This includes two genes encoding cysteine proteases: *CP1 *(242.m00078) and a putative *CP *(10.m00362), lysozyme (52.m00148) and a gene encoding the 35 kDa subunit of the amebic Gal/GalNAc lectin (17.m00351). Several of these genes have previously been identified as being trophozoite-specific, thus their down regulation is a further indication of the transcriptional activation of the encystation pathway in TSA-treated parasites [14].

### Genomic regions controlled by histone acetylation

We investigated whether there were genomic regions containing multiple genes that were regulated by TSA. Such regions may be indicative of regions where gene expression is regulated by chromatin structure. We identified a cluster of three genes on scaffold 123 (123.m00113, 123.m00122, and 123.m00123) that were all upregulated by TSA. Additionally, a large cluster of genes strongly down regulated by TSA was observed on scaffold 223 (223.m00067, 223.m00068, 223.m00069, 223.m00070, 223.m00071, 223.m00074, 223.m00075, 223.m00076, 223.m00077, 223.m00078 and 223.m00079). The 223 chromosomal region had also been identified as being enriched for trophozoite-specific genes [14]. Whether expression from these genomic regions is repressed by histone acetylation, or whether the effect is indirect, needs to be determined experimentally.

## Discussion

Gene expression can be transcriptionally controlled by epigenetic mechanisms including DNA methylation and histone modification. In order to define the genome-wide extent of regulation of gene expression by histone modification in *Entamoeba histolytica*, we performed expression profiling of *E. histolytica *trophozoites with short chain fatty acids and Trichostatin A (both histone deacetylase inhibitors). Our results identified that in contrast to effects seen in other eukaryotic systems, and despite inducing changes in histone acetylation, SCFA induce minimal transcriptional changes in *E. histolytica *trophozoites. However, the parasites do modulate gene expression significantly in response to TSA. The TSA induced transcriptional signature was distinct from changes induced by inhibition of DNA methylation but strongly overlapped with the gene expression profile of encystation in *E. histolytica*.

*E. histolytica *trophozoites normally grow and differentiate in the presence of SCFA while they reside in the lumen of the colon. SCFA are known to regulate gene expression in colonic epithelial cells which are normally exposed to SCFA [29-32]. When *Entamoeba *parasite isolates are initially collected from infected individuals, the trophozoites are cultured with the accompanying bacteria, which produce SCFA. Subsequently, *E. histolytica *isolates are selected for an ability to grow in medium that does not contain bacteria or SCFA. As only a small number of genes changed expression levels in response to SCFA, either the axenic parasites have lost nearly all of their transcriptional response to SCFA, or these compounds do not normally exert a large influence on gene expression at the transcriptional level in parasites *in vivo*. SCFAs do inhibit encystment, however, and based on the described results here, this may be occurring via more subtle changes in transcript levels (that did not meet the fold-change criteria applied to the data) or more likely through post-transcriptional mechanisms.

In contrast to SCFA, treatment of *E. histolytica *200:NIH trophozoites with TSA demonstrated changes in gene transcript levels. This indicates that when class I/II HDAC enzymes are specifically targeted in *Entamoeba *and increased amounts of histone hyperacetylation occur [19], transcriptional changes follow. Like other eukaryotic cells, then, the expression of a small fraction of the genome of *Entamoeba *parasites appears to be sensitive to hyperacetylation of core histones. Transcriptional profiling was previously performed on *E. histolytica *parasites treated with 5-azacytidine (5-AzaC), an inhibitor of DNA methyltransferase, showing that ~2.1% of genes were differentially regulated by 5-AzaC exposure [16]. There was no significant overlap between the genes found here to be regulated by TSA and those regulated by 5-AzaC. Thus, epigenetic types of regulation, including both DNA methylation and histone acetylation, do play roles in gene expression mechanisms in *E. histolytica*, but the set of genes regulated by these processes is limited and non-overlapping. This is similar to the situation in *Arabidopsis thaliana*, in which genes regulated by 5-AzaC and TSA do not overlap, although a synergistic effect of treatment with both compounds has been observed [33]. In contrast, in human carcinoma cells TSA treatment results in DNA demethylation [34], one indication of the increasing levels of complexity of the mechanisms that establish histone codes in higher eukaryotes [7].

The greater significance of the gene expression changes induced by TSA was their overlap with the transcription profile of parasites undergoing differentiation. Initially these data may seem at odds with previously published data in which addition of TSA to encysting cultures of *E. invadens *was found to block encystment [23]. However, there are several possible explanations for this result. First, here we added TSA to vegetative *E. histolytica *trophozoite stage cells, whereas previous studies tested the effects of TSA on encysting *E. invadens*, and TSA effects on trophozoites and encysting parasites may be distinct. Second, the conclusions that TSA inhibits encystation in *E. invadens *were based on its ability to prevent production of a chitin-containing cyst, the end product of the differentiation pathway. The transcriptome data, in contrast, is a more revealing assessment of induction of the differentiation pathway. In fact, no genes known to encode proteins involved in cyst wall synthesis, such as chitin synthase or the glycoprotein Jacob, were regulated by TSA. Histone acetylation may therefore play an early role in cell fate determination and not regulate genes involved in the terminal stages of differentiation. Another possibility is that *E. histolytica *and *E. invadens *have opposing responses to TSA. However, this seems unlikely given the recent observation that conditions that support encystation in *E. histolytica *also permit spontaneous encystation in *E. invadens *[14], and both species respond to TSA with similar hyperacetylation responses [19].

**Figure 4:**
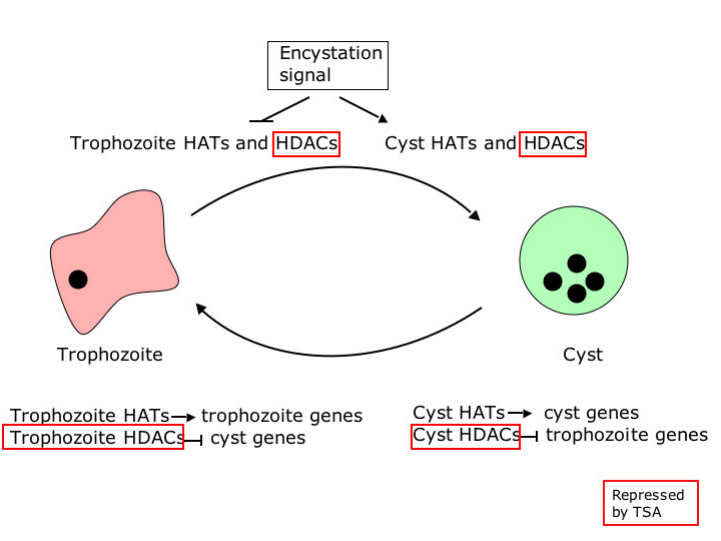
**A proposed model for the role of histone acetylation in *Entamoeba *stage conversion**. Under axenic growth conditions, trophozoite-specific HATs and HDACs induce the expression of trophozoite genes while suppressing the expression of cyst genes. When TSA is added to these cultures, the repression of HDAC activity allows expression of cyst genes. In contrast, during encystation cyst-specific HATs and HDACs become active, turning off trophozoite-specific genes and inducing cyst genes. TSA treatment of cells induced to encyst may repress the activity of cyst-specific HDACs, allowing continued expression of trophozoite genes and blocking completion of the encystation pathway. Steps sensitive to TSA treatment are highlighted in red.

Another model to consider is based on results from *Toxoplasma gondii*, in which the expression of both cyst and tachyzoite-specific genes is regulated by histone acetylation states [24]. This finding implied the existence of HATs and HDACs whose activities are developmentally regulated. If a similar situation were also the case in *Entamoeba*, trophozoite-specific HDAC activity would normally block activation of cyst-specific genes in trophozoites, and this block would be lifted upon TSA treatment, leading to the observed expression of a fraction of the cyst-specific genes. However, TSA treatment after induction of the encystation program would repress cyst-specific HDACs and inhibit the repression of trophozoite-specific genes, hence arresting the encystation program (Figure 4). Overall, the overlap between TSA-induced and cyst-specific genes and the overlap between TSA-repressed and trophozoite-specific genes is strong evidence that histone acetylation states are part of the mechanisms that regulate developmental pathways in *E. histolytica*.

## Conclusion

We have used whole-genome expression profiling to demonstrate that *E. histolytica *200:NIH trophozoites have dichotomous responses to SCFA and TSA, both histone deacetylase inhibitors. Despite affecting changes in histone acetylation, and in contrast to data from other eukaryotic systems, short chain fatty acids induce minimal transcriptional changes in *E. histolytica *trophozoites. In contrast, TSA has both a significant effect on histone acetylation and induces transcriptional changes. Importantly the transcriptional pathway modulated by TSA overlaps significantly with the gene expression changes seen with developmental conversion from trophozoites to cysts. This work identifies for the first time a molecular signature of TSA effects on *E. histolytica *parasites and lays the groundwork for further dissection of the roles of histone acetylation on amebic development.

## Methods

### *Entamoeba *strains and culture methods

The strains used in this study were *E. histolytica *HM-1:IMSS and *E. histolytica *200:NIH both of which were grown axenically in TYI-S-33 medium under standard culture conditions at 36.5°C [35]. Additionally, both strains were grown in TYI-S-33 medium in the absence of glucose (LG medium), in TYI-S-33 medium plus SCFA (70 mM sodium acetate, 20 mM sodium propionate and 10 mM sodium butyrate) (SCFA medium) [23], and in TYI-S-33 medium plus TSA (150 nM or 300 nM) (TSA medium). Genotypes of the *E. histolytica *strains (HM-1:IMSS and 200:NIH) were confirmed by PCR and RFLP based on previously published methods [36,37].

### RNA isolation and microarray hybridization

Total RNA was isolated using Trizol reagent (Invitrogen) using the manufacturer's protocol and purified using a Qiagen RNeasy kit before being used for microarray analysis [14]. Samples were processed for microarray hybridization by the Stanford University Protein and Nucleic Acids facility [38] using standard protocols. For each sample the RNA quality was checked using an Agilent BioAnalyzer QC and 4 μg subjected to the standard labeling and hybridization method [39]. *E. histolytica *200:NIH parasites were grown in SCFA for 16 hours, harvested and RNA extracted for microarray experiments. *E. histolytica *200:NIH parasites were grown in TSA (150 nM or 300 nM) for 16 or 72 hours (150 nM), harvested and RNA extracted for microarray experiments.

Labeled samples were hybridized to a custom generated Affymetrix platform full genome microarray (E_his-1a520285F), which has been previously described [27]. This array has 7,712 unique probe sets, which represent 9,435 open reading frames. Due to the highly repetitive nature of the *E. histolytica *genome, some of the probe sets are predicted to cross-hybridize with other sequences. Probe sets that represent a single gene and do not cross hybridize are labeled as (_at). Probe sets in which at least one probe may cross-hybridize with another gene(s) are labeled as (_x_at). In situations where all the probes for a given gene cross-hybridize with another gene(s), the probe sets is labeled as (_s_at) and additional genes that cross-hybridize with this probe set are listed in Additional File 3B. This array also contains probes for intergenic non-coding regions, however, these probe sets were excluded from all analysis. After hybridization, arrays were scanned and probe intensities calculation using Affymetrix GCOS software [40].

### Microarray data normalization and analysis

Normalized expression values for each probe set were obtained from raw probe intensities in R 2.2.0 downloaded from the BioConductor project [41], using robust multi-array averaging with correction for oligo sequence (gcRMA) [42]. To identify differentially expressed genes, we used local pooled error testing [43] along with Benjamini-Hochberg multiple test correction [44]. In addition, fold-change was calculated in Genespring GX [45]. A minimum of three arrays from each condition were used for analysis of SCFA or TSA effects. Correlation coefficients were calculated in Genespring using standard correlation. Probe sets were considered differentially expressed between two conditions if they had at least a 2-fold change and were significant with a false discovery rate (FDR) of < 0.05, and were identified as "present" in at least one array. Datasets of transcriptional profiles from *E. histolytica *HM-1:IMSS, *E. histolytica *Rahman, parasites from an *in vivo *model of colitis, encystation, and 5-AzaC treatment were obtained from previously published data [14,16,27].

### Semi-quantitative reverse transcriptase polymerase chain reaction (RT-PCR)

*E. histolytica *200:NIH trophozoites grown in TYI-S-33 LG medium were transferred in mid log phase (3 × 10^5 ^per ml) into TYI-S-33 LG or TYI-S-33 LG/150nM TSA and incubated for 16 hrs. Total RNA was isolated with RNAzol, and 2ug RNA was treated with DNAase I for 5 minutes at 37°C. cDNA was synthesized with oligo-dT and Superscript III reverse transcriptase (Invitrogen) at 50°C for 2 hr. Ten-fold dilutions of cDNA were used as template for 30 cycles of PCR amplification with gene-specific primers. PCR products were fractionated on 1.5% agarose gels, stained with ethidium bromide, and photographed with a GE/Amersham ImageQuant ECL recorder. Primers used in the study are:

135.m00113 Sense (5'-CCGAATCTGCATTTCCAACT-3') and

135.m00113 Antisense (5'-CAATCCCTCCTCCAAGTGAA-3');

135.m00113 Sense (5'-TCTACTTGGAGGAGGGATTC-3') and

135.m00113 Antisense (5'-AATGAATTTGCATTGCATGG-3');

14.m00310 Sense (5'-GCCAGTTTCATTCCATGGTT-3') and

14.m00310 Antisense (5'-TCAGGACCACCAACATTTGA-3');

337.m00049 Sense (5'-TCAATGAATTGGTCGTTTGC-3') and

337.m00049 Antisense (5'-TCGTTTTGGTGTGAAATGTTG-3');

146.m00117 Sense (5'-CCCCATCCAAAATTGAACAG-3') and

146.m00117 Antisense (5'-GGATGGGGATTAGAAACCAAA-3');

223.m00071 Sense (5'-CCTAAACTTCAGCAAGTTCATTCA-3') and

223.m00071 Antisense (5'-GAAAGAAGTTGAGCCCAAAGCA-3');

1.m00712 Sense (5'-AACAATTGGTCAATGCTTCTCA-3') and

1.m00712 Antisense (5'-TCCCAAATGAACGAATAGGC-3');

223.m00075 Sense (5'-TGCAAAAATTAATAACCTTCTTCG-3') and

223.m00075 Antisense (5'-TCCACCAACAAAACCTGAAA-3');

77.m00173 Sense (5'-CAACATCTATTGGAAAAAGACCA-3') and

77.m00173 Antisense (5'-TGGAGATAACTCCTTCTCCATCA-3');

340.m00050 Sense (5'-CATCGAATATGATATTACATCAAATG-3') and

340.m00050 Antisense (5'-TTTATTGGAATTGGGTCAATAGCATTC-3');

247.m00075 Sense (5'-TGCAAAGTCCATTTCCAACA-3') and

247.m00075 Antisense (5'-TTTCAGGAGAAAAAGTGGCTTC-3');

7.m00480 Sense (5'-TGATTGCAAAAGATTCAGAAACA-3') and

7.m00480 Antisense (5'-ACTTGACCCAAAGTCATCACG-3');

13.m00291 Sense (5'-TGCTCAATGGCATCAATGTT-3') and

13.m00291 Antisense (5-'GCTTCCATTTGGGACGTAGA-3');

ssRNA Sense: (5'-ACGAACGAGACTGAAACCTAT-3') and

ssRNA Antisense: (5'-TGTTACGACTTCTCCTTCCTC-3').

## Abbreviations

TSA: Trichostatin A; HDAC: histone deacetylases; RT-PCR: reverse transcriptase polymerase chain reaction; SCFA: short chain fatty acids; 5-AzaC: 5-Azacytidine; FDR: false discovery rate.

## Authors' contributions

GME, US and DJE designed the experiments, GME and DJE performed the experiments, and GME, US, and DJE wrote the manuscript. All authors have read the final version of the manuscript and agree with its contents.

## Supplementary Material

Additional file 1 **Genes differentially expressed in *E. histolytica *HM-1:IMSS, *E. histolytica *Rahman, and *E. histolytica *200:NIH strains. (A) **Genes differentially expressed in *E. histolytica *HM1:IMSS compared to *E. histolytica *Rahman and 200:NIH. The TIGR gene number, annotation, and fold-change are shown. Normalized expression data for *E. histolytica *HM-1:IMSS and Rahman strains grown in TYI-S-33 media were obtained from previously published literature [14] and subjected to local pooled error testing along with Benjamini-Hochberg multiple test correction. Genes that have higher expression in *E. histolytica *HM-1:IMSS than in 200:NIH or Rahman are listed under "up". Genes that have lower expression in *E. histolytica *HM-1:IMSS than in 200:NIH or Rahman are listed under "down". **(B) **Genes differentially expressed in *E. histolytica *Rahman compared to *E. histolytica *200:NIH and HM-1:IMSS. The TIGR gene number, annotation, and fold-change are shown. Genes that have higher expression in *E. histolytica *Rahman than in HM-1:IMSS or 200:NIH are listed under "up". Genes that have lower expression in *E. histolytica *Rahman than in HM-1:IMSS or 200:NIH are listed under "down". **(C) **Genes differentially expressed in *E. histolytica *200:NIH compared to *E. histolytica *Rahman and HM-1:IMSS. The TIGR gene number, annotation, and fold-change are shown. Genes that have higher expression in *E. histolytica *200:NIH than in HM-1:IMSS or Rahman are listed under "up". Genes that have lower expression in *E. histolytica *200:NIH than in HM-1:IMSS or Rahman are listed under "down". Click here for file

Additional file 2 **Expression profiles of histone and chromatin modifying genes in *E. histolytica *strains HM-1:IMSS, 200:NIH, and Rahman. **The mean trophozoite expression value for *E. histolytica *HM-1:IMSS, 200:NIH, and Rahman strains under standard culture conditions, GenBank ID number, and gene annotations are shown. Additional loci represented by crosshybridizing _s_at probe sets are as follows – 49.m00192_s_at: 334.m00048, 361.m00053 and 472.m00060; 100.m00145_s_at: 48.m00184; 4.m00618_s_at: 280.m00075; 9.m00405_s_at: 117.m00168 and 67.m00101; 39.m00254_s_at: 35.m00257; 444.m00042_s_at: 25.m00257. Genes that are statistically different in one strain compared to the other two are in bold. Click here for file

Additional file 3 **Data for all arrays based on MIAME format. (A) **Normalized array data for all genes. The Probe ID, TIGR gene number, GenBank accession, Annotation, fold-change, and FDR are shown. The array data for *E. histolytica *200:NIH grown in TYI-S-33 media and *E. histolytica *200:NIH exposed to low glucose (LG), Trichostatin A (TSA), and short chain fatty acids (SCFA) are listed. Normalized expression data for *E. histolytica *HM-1:IMSS and Rahman strains grown in TYI-S-33 media were obtained from previously published literature [14]. **(B) **Representation table for all _s_at probes. Click here for file

Additional file 4 **Genes differentially regulated in *E. histolytica *200:NIH by exposure to TSA media. (A) **Genes with increased expression in TSA media. **(B) **Genes with decreased expression in TSA media. The Probe ID, TIGR gene number, GenBank accession, Annotation, fold-change, and FDR are shown. All array data are available at the GEO database website at NCBI (Accession number GSE8047). Click here for file
